# Evaluation of laser power stability of repeatedly used SubCyclo probe in micropulse transscleral cyclophotocoagulation for glaucoma: A step towards sustainable ophthalmic surgery

**DOI:** 10.1371/journal.pone.0295517

**Published:** 2023-12-08

**Authors:** Pukkapol Suvannachart, Ploysai Rujkorakarn, Thanita Watha, Parinya Srihatrai

**Affiliations:** Department of Ophthalmology, Suddhavej Hospital, Faculty of Medicine, Mahasarakham University, Maha Sarakham, Thailand; Chiemsee Augen Tagesklinik, Technical University of Munich, GERMANY

## Abstract

**Purpose:**

To evaluate the laser power stability of the SubCyclo probe for micropulse transscleral cyclophotocoagulation after repeated use.

**Materials and methods:**

This experimental study involved 6 new probes. Each probe was connected to the SubCyclo mode (2,000 mW power, 31.3% duty cycle, and 100 seconds duration) of the Vitra 810 laser delivery system (Quantel Medical, France). Laser power measurements were taken using a calibrated laser power meter (Nova, Ophir Optronics Solutions, Israel) every 10 seconds from 10 to 90 seconds during each of the 40 cycles. Intra-rater reliability was assessed using intraclass correlation (ICC). A linear mixed model for repeated measures and pairwise comparisons with Bonferroni adjustment were used for the analysis.

**Results:**

The mean (SD) power outputs of all probes for the first cycle and all cycles were 421.9 (19.7) mW and 436.7 (16.1) mW, respectively. During the first cycle, the mean (SD) laser power gradually decreased from 444.3 (13.4) mW at 10 seconds to 407.3 (17.0) mW at 90 seconds (Fig 3). For all cycles, the power was 446.0 (13.6) mW at 10 seconds and gradually declined to 426.8 (21.0) mW at 90 seconds. Pairwise comparisons revealed significant differences in mean laser power outputs after 16 cycles of repeated use compared to the first cycle. The ICC estimate (95% CI) for intra-rater reliability was 0.96 (0.89, 0.99).

**Conclusions:**

The SubCyclo probe maintains stable laser power outputs throughout repeated use for up to 16 cycles, with a significant increase observed after 16 cycles.

## Introduction

Micropulse transscleral cyclophotocoagulation (MP-CPC) is a safe and effective procedure for reducing intraocular pressure (IOP) in different types and severities of glaucoma [1, 2]. The on-and-off technology used in MP-CPC avoids adjacent tissue damage thus provides better safety compared to conventional continuous wave diode laser transscleral cyclophotocoagulation (CW-CPC) [3]. During MP-CPC, diode laser energy is transmitted through a fiber-optic probe transsclerally and intermittently to the underlying ciliary body. The disposable probe is available in an individual package which is pre-sterilized using ethylene oxide from the factory. Several models of the micropulse laser delivery device and their compatible probes are available, including the Cyclo G6 laser system with Micropulse P3 probe (Iridex, CA), and the Vitra 810 laser system with SubCyclo probe (Quantel Medical, France). The SubCyclo probe has a removable footplate that allows its use in both MP-CPC and CW-CPC.

Climate change is a long-term alteration of temperatures and weather patterns resulting from excessive greenhouse gas emissions. This crisis is an ongoing problem that is affecting the entire ecosystem. Health care was estimated to release approximately 4.4% of total greenhouse gas emissions worldwide [4]. As clinicians have tended to use more disposable items over the past two decades, disposable items now account for up to 20 times as much waste as reusable instruments [5]. In addition to their environmental effects, disposable products also result in significant financial costs for health systems and patients. The “three Rs”—reduce, reuse, and recycle—provide a simple approach to help reduce waste and costs from the health care sector, including ophthalmic surgery. The use of reusable surgical gowns and instruments, along with paper waste recycling, can also lead to waste and cost reduction.

Most cyclophotocoagulation probes are recommended for single use by manufacturers, and each probe costs approximately 280 USD. Although reusing probes could be a cost-effective way to decline costs and excessive amount of waste produced from surgery, it is currently off-label in most countries. However, several studies have investigated the possibilities for reusing probes, and the current evidence is limited and demonstrates mixed results. The disposable G-probe (Iridex, CA) for CW-CPC was still functional with some changes in power output after repeated use [6, 7]. The Micropulse P3 probe, which is activated for 90 minutes after use, showed an average decrease of 3% in laser energy delivered per repeated cycle [8]. In contrast, the SubCyclo probe has no time limitation, but there are no studies to support its power stability upon reuse.

Before adopting probe reuse, three key considerations must be addressed. First, the probe’s ability to maintain stable laser power output after repeated use should be established. Second, a standardized cleaning and resterilization protocol needs to be defined, considering its impact on power changes. Finally, comprehensive studies are required to evaluate the safety and effectiveness of reused probes in clinical settings. This study aims to evaluate the laser power stability of the SubCyclo probe after repeated use.

## Materials and methods

The Ethics Committee of Mahasarakham University granted an exemption for this study (approval number: 024-099/2021), and informed consent was waived since the study did not involve human subjects. This experimental study utilized six unused SubCyclo probes. All probes were examined for any defects under a slit lamp biomicroscope with high magnification (40x) before the study and after all measurements.

### Laser power setting

The Vitra 810 (Quantel Medical, France) was used to generate 810-nanometer wavelength laser for the treatment of glaucoma with the SubCyclo mode. The SubCyclo probe was connected to the device, and the power setting was adjusted to 2,000 milliwatts (mW) with a duty cycle of 31.3%. The estimated power delivery with this setting was 626 mW. The duration for each cycle was 100 seconds. A total of 40 cycles were performed for each probe during the experiment.

### Measurement protocol

**Fig 1:**
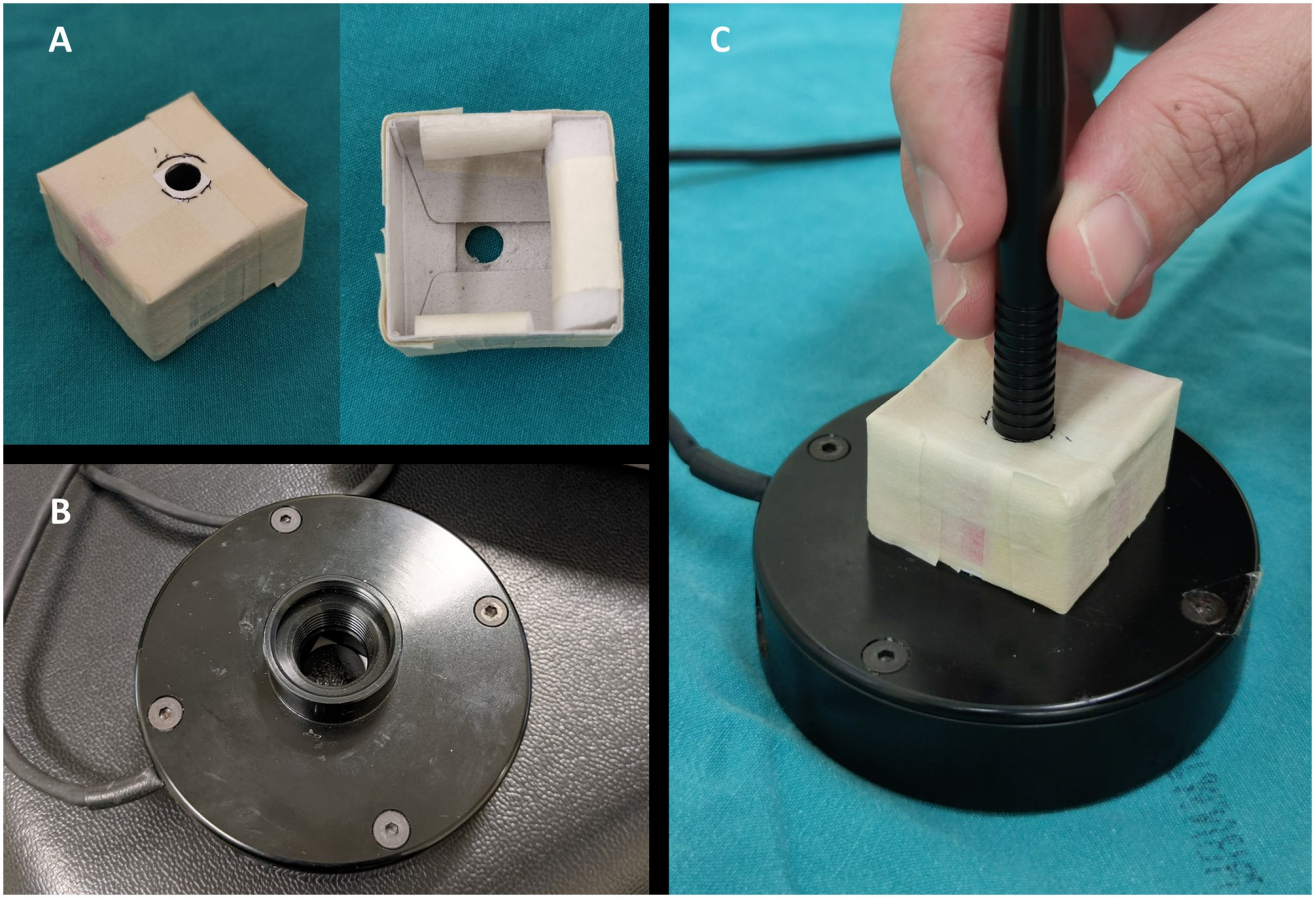
Custom-made probe stand. (A) Constructed from a paper box and sponge, (B) 3A-P laser power sensor, (C) Measurement using the probe stand.

The measurement of laser power output was carried out by one glaucoma specialist (PS), using a regularly calibrated Nova laser power meter (Ophir Optronics Solutions, Israel) connected to a 3A-P sensor (Ophir Optronics Solutions, Israel). The sensor is capable of measuring power ranging from 15 microwatts to 3 Watts (W) with the accuracy varying up to 3 percent. For each cycle, the power was continuously fired until the cycle was completed. Laser power outputs were recorded every 10 seconds from 10 to 90 seconds by another investigator (PR), resulting in a total of 40 cycles for each probe. To maintain a consistent position between the probe and the sensor, a custom-made probe stand, constructed from a paper box and sponge, was used (Fig 1). The probe tip was positioned approximately 5 millimeters from the sensor area without the use of any additional media during the measurement. The average of nine readings for each cycle represented the cycle’s power output.

### Statistical analyses

All statistical analyses were performed using IBM SPSS statistics for Windows version 26 (IBM Corp, NY). Descriptive statistics, including mean and standard deviation (SD), were used based on the distribution. The intraclass correlation (ICC) estimate and the 95% confidence interval (95% CI) for the intra-rater reliability of the SubCyclo probe were calculated based on a mean-rating (k = 9), absolute agreement, and 2-way mixed effects model. ICC estimate was interpreted using the classification proposed by Koo and Li [9]. Laser power output was analyzed using linear mixed models to account for the correlations between repeated measurements within each probe, with restricted maximum likelihood estimation. The model used a compound symmetry covariance matrix for repeated measures and included fixed effects of cycle. Pairwise comparisons with Bonferroni adjustment were conducted to analyze the differences between the first cycle and subsequent cycles. A p-value of less than 0.05 was considered statistically significant.

## Results

**Fig 2:**
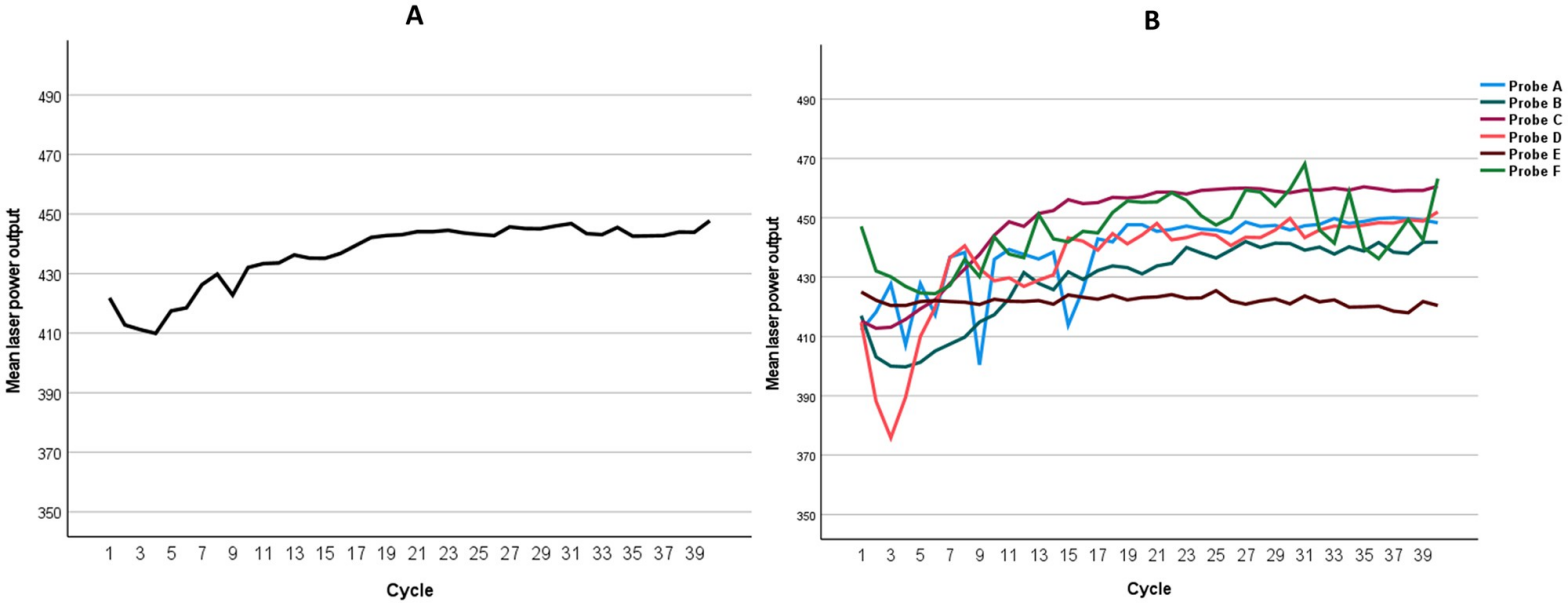
Laser power outputs following repeated use. (A) All probes, (B) Each probe.

**Fig 3:**
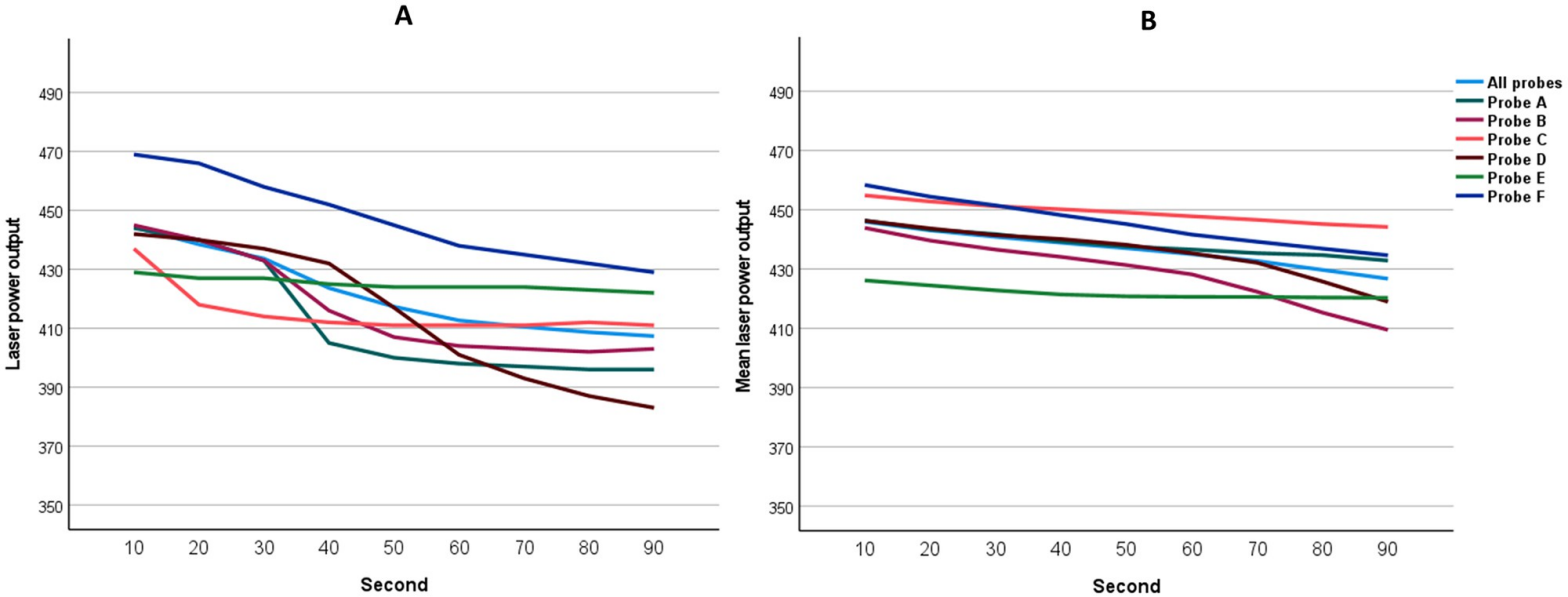
Laser power outputs at different timepoints. (A) 1^st^ cycle, (B) All cycles.

All six probes were unused and appeared normal under the biomicroscope before the study. For each probe, 40 cycles of laser delivery were performed. The mean (SD) power outputs of all probes for the first cycle and all cycles were 421.9 (19.7) mW and 436.7 (16.1) mW, respectively (Table 1). Linear plots of mean laser power outputs following repeated use are shown in Fig 2. For the first cycle, the mean (SD) laser power gradually decreased from 444.3 (13.4) mW at 10 seconds to 407.3 (17.0) mW at 90 seconds (Fig 3). For all cycles, the power was 446.0 (13.6) at 10 seconds and gradually declined to 426.8 (21.0) mW at 90 seconds (Fig 3). The power outputs for each timepoint are also shown in Table 1.

**Table 1:** Laser power outputs following repeated use.

	Overall	Probe A	Probe B	Probe C	Probe D	Probe E	Probe F
**1**^**st**^ **cycle (reference cycle), mean (SD) in mW**							
10 seconds	444.3 (13.4)	444	445	437	442	429	469
20 seconds	438.5 (16.2)	440	440	418	440	427	466
30 seconds	433.7 (14.4)	433	433	414	437	427	458
40 seconds	423.7 (16.8)	405	416	412	432	425	452
50 seconds	417.3 (15.9)	400	407	411	417	424	445
60 seconds	412.7 (15.5)	398	404	411	401	424	438
70 seconds	410.5 (16.3)	397	403	411	393	424	435
80 seconds	408.7 (16.9)	396	402	412	387	423	432
90 seconds	407.3 (17.0)	396	403	411	383	422	429
1^st^ cycle power	421.9 (19.7)	412.1 (20.5)	417.0 (17.5)	415.2 (8.5)	414.7 (24.0)	425.0 (2.2)	447.1 (14.9)
**All cycles, mean (SD) in mW**							
10 seconds	446.0 (13.6)	446.4 (7.1)	443.9 (9.3)	454.9 (12.0)	446.3 (8.3)	426.2 (1.9)	458.4 (12.1)
20 seconds	443.1 (15.3)	443.3 (10.3)	439.6 (13.2)	452.8 (14.2)	443.7 (13.9)	424.5 (2.2)	454.45 (12.6)
30 seconds	440.9 (16.3)	441.8 (12.5)	436.6 (15.4)	451.2 (15.4)	441.5 (16.1)	422.8 (2.3)	451.5 (12.4)
40 seconds	438.9 (17.5)	439.4 (15.6)	434.1 (17.4)	450.2 (16.4)	440.2 (18.4)	421.4 (2.3)	448.2 (12.6)
50 seconds	437.1 (18.0)	437.6 (17.2)	431.4 (18.2)	449.1 (17.2)	438.2 (19.1)	420.8 (2.2)	445.2 (12.9)
60 seconds	435.1 (18.0)	436.6 (15.8)	428.2 (18.3)	447.8 (17.7)	435.4 (21.2)	420.6 (1.9)	441.7 (12.7)
70 seconds	432.7 (18.5)	435.4 (16.6)	422.4 (17.8)	446.6 (18.2)	432.1 (22.8)	420.6 (1.8)	439.2 (12.1)
80 seconds	429.7 (19.6)	434.7 (16.5)	415.3 (16.5)	445.2 (18.5)	425.8 (26.3)	420.4 (1.7)	436.9 (10.9)
90 seconds	426.8 (21.0)	432.9 (18.8)	409.5 (13.1)	444.2 (18.8)	419.0 (29.5)	420.3 (2.1)	434.7 (10.2)
All cycle power	436.7 (16.1)	438.7 (13.3)	429.0 (13.7)	449.1 (15.8)	435.8 (17.7)	422.0 (1.6)	445.6 (11.3)

Pairwise comparisons demonstrated significant differences in mean laser power outputs after 16 cycles of repeated use compared to the first cycle (Table 2). Beyond this point, laser power outputs exhibited a significant increase compared to the baseline cycle, with an approximate range of 17.6 to 25.9 mW (Table 2). The ICC estimate (95% CI) for intra-rater reliability of the SubCyclo probes was 0.96 (0.89 to 0.99). After all measurements, all probes were examined and revealed no physical defects.

**Table 2:** Pairwise comparisons of laser power output between cycles.

Cycle (I)	Reference cycle (J)	Mean differences (I-J)	95% CI for mean differences		P-value
			Lower bound	Upper bound	
2	1	-9.1	-25.0	6.8	1.000
3	1	-10.6	-26.6	5.3	1.000
4	1	-12.0	-27.8	4.0	0.599
5	1	-4.4	-20.3	11.5	1.000
6	1	-3.3	-19.2	12.6	1.000
7	1	4.4	-11.5	20.3	1.000
8	1	8.0	-7.9	23.9	1.000
9	1	1.0	-14.9	16.9	1.000
10	1	10.2	-5.7	26.1	1.000
11	1	11.5	-4.4	27.4	0.740
12	1	11.7	-4.2	27.7	0.656
13	1	14.4	-1.5	30.3	0.134
14	1	13.4	-2.6	29.3	0.260
15	1	13.3	-2.6	29.2	0.269
16	1	15.0	-1.0	30.8	0.097
17	1	17.6	1.7	33.5	0.015*
18	1	20.3	4.4	36.2	0.002*
19	1	21.0	5.0	36.9	0.001*
20	1	21.2	5.3	37.1	<0.001*
21	1	22.3	6.3	38.2	<0.001*
22	1	22.2	6.3	38.2	<0.001*
23	1	22.7	6.8	38.6	<0.001*
24	1	21.8	5.9	37.7	<0.001*
25	1	21.3	5.4	37.3	<0.001*
26	1	20.9	5.0	36.8	0.001*
27	1	23.9	7.9	39.8	<0.001*
28	1	23.3	7.4	39.2	<0.001*
29	1	23.2	7.3	39.1	<0.001*
30	1	24.2	8.3	40.1	<0.001*
31	1	25.0	9.1	40.9	<0.001*
32	1	21.6	5.7	37.5	<0.001*
33	1	21.2	5.3	37.2	<0.001*
34	1	23.7	7.8	39.6	<0.001*
35	1	20.7	4.8	36.6	0.001*
36	1	20.8	4.9	36.7	0.001*
37	1	20.9	5.0	36.8	0.001*
38	1	22.1	6.2	38.0	<0.001*
39	1	22.1	6.1	38.0	<0.001*
40	1	25.9	1.0	41.8	<0.001*

*The mean difference is significant at a level of less than 0.05 level

## Discussion

Healthcare carbon emissions have multiple sources, including energy consumption and single-use products from medication and surgery. One phacoemulsification procedure in the United Kingdom can produce around 181.8 kilograms of carbon dioxide, which is equivalent to nearly 700 kilometers of a car driving [10]. Nevertheless, the same operation in India, using reusable instruments and materials, generated only 6 kilograms of gases with comparable patient outcomes and significant cost reduction [11]. As single-use products have no proven benefit in postoperative endophthalmitis risk [12], reusable products should also be applied to other ophthalmic procedures to improve cost-effectiveness and overall carbon footprint.

Both CW-CPC and MP-CPC probes, including the SubCyclo probe, are disposable. Since MP-CPC is relatively non-invasive, probe reuse could be an eco-friendly, safe, and cost-effective approach. However, ensuring patient safety requires evidence on several aspects, including the maintenance of laser power stability after repeated use, the establishment of a standardized cleaning and resterilization protocol, and an evaluation of the safety and effectiveness in clinical use. In our routine practice for MP-CPC, we generally perform 2 cycles of this setting (2,000 mW power, 31.3% duty cycle, and 100 seconds duration) on upper and lower quadrants for most patients. The total power of 125.2 Joules was within the safe range (112 to 150 Joules) [13]. The average power readings, ranging from 436.7 mW as shown in Table 1, were lower than the estimated power output from the setting (626 mW) by up to 30.2%. This difference could be explained by the absence of contact medium. Patel et al. found a significantly lower mean power (358 mW) from estimated power (626 mW) in the absence of contact medium for Micropulse P3 probes [14].

In our study, we selected forty cycles based on the estimated cost-effectiveness and potential waste reduction in our specific setting. While demonstrating excellent intra-rater reliability, as indicated by the ICC estimate, SubCyclo probes maintained stable power outputs for up to 16 cycles. Beyond this point, laser power outputs exhibited a significant increase compared to the baseline cycle (Table 2). Although several studies evaluated laser power output following repeated use of different fiber-optic cyclophotocoagulation probes, none were done with the SubCyclo probe. The G-probe for CW-CPC had conflicting results. Tham et al. found an average decrease of 3% in laser energy delivered per repeated cycle [6]. In contrast, Carrillo et al. found an average energy increase of approximately 2 mW per cycle [7]. The Micropulse P3 probe for Cyclo G6 MP-CPC showed similar results to our study, suggesting a potential increase in laser output with repeated use, especially after 4 cycles [8]. Our findings are likely attributed to the inherent characteristics of the SubCyclo probe, as we conducted all measurements continuously without employing a sweeping technique or cleaning process. Other factors, such as the accumulation of debris, surface abrasion resulting from a sweeping motion during the cycle, and the cleaning process, could potentially influence laser output changes, which might introduce interference with the results as suggested in previous studies.

Interestingly, as we measured at 9 different time points within each cycle, we observed a gradual change in laser power output during continuous firing, with higher power recorded at the earlier time points of each cycle for all probes (Fig 2). This finding may suggest that avoiding pauses during the procedure is advisable when treating patients and designing research protocols. Such pauses may inadvertently lead to higher total power being delivered to patients and artificially elevated laser power output measurements during research.

To our knowledge, this is the first study to evaluate the laser power stability of the SubCyclo probe. Although our study showed that the SubCyclo probe had stable laser power for up to 16 cycles, it is important to note that the results of this study alone are not sufficient to support routine probe reuse in clinical practice. Instead, they serve as a starting point for further research and exploration of probe reuse. Future experiments with a defined protocol for the cleaning and resterilization process after each use would enable a more comprehensive investigation of the factors influencing laser power outputs in resterilized probes. This would ultimately contribute to the development of a more robust and standardized protocol for the future reuse of probes, laying the foundation for potential clinical trials in the future.

## Conclusion

The SubCyclo probe maintains stable laser power outputs throughout repeated use for up to 16 cycles, with a significant increase observed after 16 cycles.

## Supporting information

S1 File Data. (XLSX)
